# Latent profile analysis of dietary behavior in pediatric patients with short stature: a single-center study in China

**DOI:** 10.3389/fpubh.2026.1919578

**Published:** 2026-09-18

**Authors:** Meng Lin, Xin Yang

**Affiliations:** 1 Department of Pediatric Genetics, Metabolism and Endocrinology Nursing, West China Second University Hospital, Sichuan University, Chengdu, Sichuan, China; 2 Key Laboratory of Birth Defects and Related Diseases of Women and Children, Ministry of Education, Sichuan University, Chengdu, Sichuan, China

**Keywords:** cross-section study, dietary behavior, latent profile, pediatric patients, short stature

## Abstract

**Objective:**

To identify potential subtypes of dietary behavior in pediatric patients with short stature using latent profile analysis (LPA) and to explore the factors associated with each latent profile.

**Methods:**

A convenience sample of 308 short-statured pediatric patients was recruited from the outpatient department of Sichuan University of West China Second Hospital from July 2025 to March 2026. Participants completed questionnaires, including a general information questionnaire, the Children’s Dietary Behavior Questionnaire and the Children’s Depression Inventory. LPA was used to identify the optimal number of subgroups (profiles), and binary logistic regression analysis was used to explore variables associated with profile membership.

**Results:**

A total of 308 valid questionnaires were collected, yielding an effective response rate of 94.77%. This study employed LPA and identified two distinct eating patterns among pediatric patients with short stature: healthy-adaptive eating (47.73%, *n* = 147) and emotion-inhibited eating (52.27%, *n* = 161). Binary logistic regression analysis revealed that age (OR = 0.728, *p* < 0.001), Height SDS (OR = 0.738, *p* = 0.030), and lack of pleasure (OR = 1.239, *p* < 0.001) were independent influencing factors of emotion-inhibited eating.

**Conclusion:**

This study revealed two distinct subtypes of dietary behaviors among pediatric patients with short stature. A lack of pleasure was an independent risk factor for emotion-inhibited eating. Healthcare professionals should pay particular attention to children’s emotional states and develop targeted interventions to address maladaptive eating patterns in this population.

## 1 Introduction

Short stature is a common pediatric endocrinological disorder in which the height is 2 standard deviations below the mean for age, sex, and ethnicity, without an identifiable cause (1). According to the literature, the prevalence of short stature among pediatric patients worldwide varies by region and population characteristics, reaching as high as 10.2% in developing countries (2). In China, approximately 3.2% of children are affected by short stature. The prevalence rate is approximately 4.7% in rural areas, whereas it is approximately 2.8% in urban areas (3, 4). As a critical period for physical and psychological development, the school-age stage is characterized by a departure from the normal height growth trajectory that may not only compromise physical development but also trigger a cascade of subsequent psychological and behavioral problems (5). Previous studies have indicated that pediatric patients with short stature often experience diminished self-esteem, social withdrawal, and internalized problems such as anxiety and depression, which may, in turn, affect their dietary behavior and nutritional intake (6).

Dietary behavior is among the key modifiable factors influencing children’s growth and development. The school-age period represents a critical phase during which dietary behaviors are formed and consolidated, and these established patterns tend to persist into adulthood, resulting in long-term effects on lifelong health (7). However, the characteristics of dietary behavior among pediatric patients with short stature have not yet been fully studied. Rather, the existing research on children’s dietary behavior has largely focused on obese children, with relatively limited attention given to the specific population of pediatric patients with short stature (8–11). More importantly, traditional dietary behavior studies have predominantly adopted a variable-centered analytical paradigm, which describes group averages by calculating total scale scores or mean scores across dimensions while overlooking the potential heterogeneity in dietary behavior patterns among individuals.

In fact, the dietary behaviors of pediatric patients with short stature with short stature may exhibit a variety of different patterns. Some children may exhibit poor appetite and inadequate food intake, others may display picky eating or food fastidiousness, and still others may consume sufficient quantities of foods that have poor dietary quality (5). Identifying these potential subtypes of dietary behavior is crucial for developing targeted nutritional interventions (12). However, research employing a person-centered approach to identify patterns in the dietary behaviors of pediatric patients with short stature is lacking.

Latent profile analysis, as an individual-centered analytical method, has been increasingly applied in the fields of public health and psychology (13), as it can partition heterogeneous populations into several homogeneous latent subgroups based on individuals’ response patterns across multidimensional observed indicators, thereby revealing structured differences within the population (14). This method not only helps in understanding the heterogeneous characteristics of dietary behavior but also provides empirical evidence for the development of stratified intervention strategies.

Furthermore, it is worth noting that there is a close interplay between eating behaviors and mental health. Research has indicated that unhealthy eating patterns are positively correlated with depressive symptoms in children (5). For pediatric patients with short stature who may face greater psychosocial stress because of physical limitations, the relationship between these eating behaviors and depression may be even more complex. However, whether there are significant differences in depression levels among pediatric patients with short stature who exhibit different eating patterns remains unclear and warrants further research.

Therefore, this study aims to (1) identify latent subtypes of dietary behavior among pediatric patients with short stature using LPA based on the eight dimensions of the Children’s Eating Behavior Questionnaire and (2) compare differences in demographic variables and depressive symptoms across different dietary behavior profiles. These findings will provide evidence to inform diet quality-based strategies for promoting mental health among children and addressing key evidence gaps in nutritional medicine during this pivotal developmental period, thus promoting the overall healthy development of this specific population.

## 2 Method

### 2.1 Study design

A single-center cross-sectional study was conducted. The Strengthening the Reporting of Observational Studies in Epidemiology (STROBE) guidelines were followed in this study (15).

### 2.2 Ethical considerations

This study was conducted in accordance with the Helsinki Declaration. Ethical approval was obtained from West China Second University Hospital, Sichuan University (Medical Research Ethics Approval No. 376, 2025). All participants provided written informed consent before they participated in this study.

### 2.3 Participants

By using convenience sampling, 308 outpatients who were diagnosed with short stature at West China Second Hospital, Sichuan University, from July 2025 to March 2026 were selected as the survey subjects. The inclusion criteria for the study population were as follows: (a) fulfillment of the diagnostic criteria for childhood short stature (1); (b) age ranging from 6 to 14 years; (c) availability of a primary caregiver aged 18 years or older who can operate a smartphone; and (d) provision of written informed consent and voluntary agreement to participate in the study. The exclusion criteria were as follows: (a) patients with concurrent severe complications; (b) patients with secondary neurological impairments; and (c) patients with communication or intellectual disabilities.

### 2.4 Sampling

The sample size was determined based on the following formula:

$n=\frac{Z_{1-\alpha /2}^{2}p(1-p)}{\delta^{2}}$

In this formula, *p* denotes the anticipated prevalence rate, δ denotes the allowable error, and *n* denotes the sample size. Drawing on recent domestic studies on pediatric dietary behaviors, the prevalence rate of targeted dietary behaviors among Chinese children was estimated to be 60% (16, 17). For the calculation, *p* = 60% was adopted, the significance level was set at α = 0.05, and δ was defined as 0.1 × *p*. The formula yielded an initial sample size of *n* ≈ 256. To account for potential attrition, the sample size was increased by 20%, resulting in a final sample of 308 participants. Nylund-Gibson et al. (18) argued that a sample size in the range of 300 ~ 1,000 is generally considered the most commonly used range for effectively leveraging mixed-model fit indices. The sample size of this study adhered to statistical requirements.

### 2.5 Measures

#### 2.5.1 Sociodemographic characteristics

The general information questionnaire was self-designed based on a literature review. The demographic data included sex, age, height, weight, school grade, history of preterm birth, caregiver educational level, and family income. Height and weight were measured uniformly using a medical weighing scale and a height measuring instrument with the same equipment serial number. All measuring devices were standardized and calibrated before use. Measurements were performed by nurses who had completed training in anthropometric measurement and had passed a competency assessment. All measurements were taken on the right side of the participant’s body. Body weight was recorded to the nearest 0.1 kg, and height was recorded to the nearest 0.1 cm. For height measurement, each participant underwent three repeated measurements. Before each measurement, the participants’ posture was readjusted to ensure that the difference between the maximum and minimum values of the three measurements did not exceed 0.5 cm. The final height was taken as the average of the three measurements.

#### 2.5.2 Children’s eating behavior questionnaire

The Children’s Eating Behavior Questionnaire (CEBQ) is a multidimensional, parent-reported questionnaire that was translated and validated in Chinese by Gao M et al. (19, 20). The CEBQ scale consists of 35 items with 8 subscales: enjoyment of food (4 items), food responsiveness (5 items), emotional overeating (4 items), desire to drink (3 items), satiety responsiveness (5 items), slowness in eating (4 items), emotional undereating (4 items) and food fussiness (6 items). Each item is rated on a 5-point Likert scale as follows: (1) never = 0, (2) rarely = 1, (3) sometimes = 2, (4) often = 3, and (5) always = 4. Higher scores indicate more severe problems with children’s dietary habits. The questionnaire was completed by parents based on their child’s actual situation during the previous week. The Cronbach’s α coefficient of this questionnaire is 0.74, and the questionnaire exhibits good internal consistency, concurrent validity with actual eating behavior, test–retest reliability, and stability over time.

#### 2.5.3 The children’s depression inventory

The Children’s Depression Inventory (CDI), developed by the American psychologist Kovacs in 1977, was constructed based on the Beck Depression Inventory. It is a self-report scale for assessing depressive symptoms in children and adolescents (21, 22). The CDI scale consists of 27 items across 5 subscales: negative mood (6 items), interpersonal difficulties (4 items), inefficacy (4 items), lack of pleasure (8 items), and low self-esteem (5 items). The participants were asked to select from a group of three sentences the sentence that be best represents their feelings and thoughts over the past 2 weeks. Questions were graded on a scale of 0 to 2. A score of zero indicates that there is no sign of the specific symptom, a score of one indicates that there is a moderate sign of the specific symptom, and a score of two indicates that there is an obvious sign of the specific symptom. Total scores ranged from 0 to 54, with scores ≥ 19 indicating signs of depression in this study (23). A higher total score is correlated with more intense depression symptoms. In the study by Wang J et al. (24), the Cronbach’s ɑ for this scale was reported to be 0.82 for Chinese children.

### 2.6 Data collection

Before participants completed the questionnaire, trained research team members used standardized instructions to explain the study’s purpose and significance to the respondents. The research team members distributed the parent questionnaires and child questionnaires separately. The CEBQ was completed independently by the parents of participating children based on their child’s eating behaviors over the previous week. The CDI was self-report by the children themselves under the guidance of research team members; for children who were younger or had limited reading comprehension, research team members provided standardized explanations to help them understand the item meanings, but all responses were chosen autonomously by the children. After the questionnaires were collected, the researchers checked them immediately to verify all items were answered. If items were unanswered, the patients were asked to complete them.

### 2.7 Statistical analysis

Data analyses were performed using SPSS Statistics Version 26.0 (IBM Corp., Armonk, NY, USA) and Mplus Version 8.0 (Muthén and Muthén, Los Angeles, CA, USA). This study primarily used LPA. Unlike variable-centered approaches, LPA aims to identify distinct patterns across multiple variables that occur consistently across individuals rather than focusing solely on individual variables or their interactions. Starting with a one-category model, incremental steps were taken until the optimal latent profile emerged. Using the scores from the 8 dimensions of the Children’s Eating Behavior Questionnaire as observed variables, one to three profiles were sequentially selected for analysis. The goodness of fit of the final model was assessed using the following three criteria (25, 26): (a) Information evaluation criteria: Model fit was assessed by comparing the disparities between expected and actual values using the Akaike information criterion (AIC), Bayesian information criterion (BIC), and adjusted Bayesian information criterion (aBIC). Smaller statistical values indicated better fit. (b) Classification evaluation criteria: The accuracy of classification was assessed using entropy values, which ranged from 0 to 1. 0.8 is high level, 0.6 is medium level, and 0.4 is low level. (c) Likelihood ratio tests: The fit differences between the models with k-1 and k profiles were compared using the Lo–Mendell–Rubin (LMR) and bootstrap likelihood ratio (BLRT) tests. When the *p* values for the LMR and BLRT were less than 0.05, a model with k profiles was superior to a model with k-1 profiles. The statistical methods employed included descriptive statistical calculations, i.e., means, standard deviations and percentages. The distribution normality was assessed using the Kolmogorov–Smirnov test. To examine differences among groups, nonparametric tests, such as the Mann–Whitney U test and the Kruskal–Wallis one-way ANOVA, were used for nonnormally distributed variables. Variance inflation factors (VIFs) were analyzed to assess potential multicollinearity in linear regression models. One-way analysis of variance, chi-square (χ^2^) tests, rank-sum tests, and logistic regression analysis were also used, and the significance level was set at α = 0.05; *p* < 0.05 indicated statistically significant differences.

## 3 Results

### 3.1 General information and dietary behavior scores

Of the 325 questionnaires distributed in this study, 308 were deemed valid, resulting in a response rate of 94.77%. Among the 308 children included in the study, 167 were male (54.22%), and 141 were female (45.78%). The mean age was 8.44 ± 2.02 years, the mean height was 123.11 ± 12.00 cm, and the mean weight was 24.96 ± 9.20 kg. Of the 308 children, 27.60% presented with depressive symptoms. The participants’ basic characteristics are provided in Table 1. The mean score of the Children’s Eating Behavior Questionnaire was 56.61 ± 9.79, and the scores for each dimension are presented in Table 2.

**Table 1:** General characteristics of pediatric patients with short stature (*N* = 308).

Variables	*N* (%)
Sex	
Male	167 (54.22)
Female	141 (45.78)
Age (years)	
6 ~ 11	278 (90.26)
12 ~ 14	30 (9.74)
School grade	
Kindergarten	37 (12.01)
Primary school	255 (82.79)
Middle school	16 (5.20)
Parent’s education	
Primary school or below	39 (12.66)
Junior high school	87 (28.25)
Senior high school or above	182 (59.09)
Household income (CNY)	
<3,000	23 (7.47)
3,000 ~ 6,000	86 (27.92)
>6,000	199 (64.61)
Preterm birth	
No	276 (89.61)
Yes	32 (10.39)
Depressive symptoms	
No	223 (72.40)
Yes	85 (27.60)

**Table 2:** Scores from the CEBQ in pediatric patients with short stature.

Domains	Range	Mean ± SD
Total CEBQ scores	0 ~ 140	56.61 ± 9.79
Satiety responsiveness	0 ~ 36	9.84 ± 3.19
Slowness in eating	0 ~ 36	7.27 ± 3.14
Food fussiness	0 ~ 24	11.61 ± 2.89
Food responsiveness	0 ~ 20	9.33 ± 1.58
Enjoyment of food	0 ~ 16	6.81 ± 2.07
Desire to drink	0 ~ 12	4.71 ± 1.24
Emotional undereating	0 ~ 16	5.59 ± 3.52
Emotional overeating	0 ~ 16	1.44 ± 1.87

### 3.2 Subtypes of dietary behavior in pediatric patients with short stature

To identify the best model, six models were estimated separately in this study. Table 3 provides model fit indices for every LPA model that was tested.

**Table 3:** Indicators for each latent profile of dietary behaviors in pediatric patients with short stature.

Model	AIC	BIC	aBIC	LMRT	BRRT	Entropy	Class counts
1	11112.208	11171.889	11121.144	-	-	-	-
2	10871.814	10965.067	10885.777	<0.001	<0.001	0.787	0.477/0.523
3	10786.002	10912.826	10804.992	0.2351	0.0000	0.812	0.433/0.397/0.170
4	10727.285	10887.679	10751.301	0.0225	0.0000	0.856	0.045/0.386/0.377/0.200
5	10705.165	10899.131	10734.208	0.5612	0.0000	0.831	0.263/0.113/0.390/0.188
6	10664.698	10892.234	10698.768	0.6408	0.0000	0.839	0.104/0.250/0.334/0.117/0.046/0.149

AIC, Akaike information criterion; BIC, Bayesian information criterion; aABIC, sample size-adjusted Bayesian information criterion; LMRT, Lo–Mendell–Rubin likelihood ratio test; BLRT, bootstrap likelihood ratio test.

Although AIC, BIC, and aBIC continued to decrease with an increasing number of profiles, the 2-profile model was identified as the optimal solution after comprehensive multi-dimensional evaluation. First, the 2-profile model yielded a highly significant LMRT (*p* < 0.001), indicating a significant improvement from the 1-profile to the 2-profile solution, whereas the 3-profile model did not achieve a significant LMRT (*p* = 0.2351), suggesting that adding a third profile did not significantly improve fit. Second, analysis of the entropy statistic suggests that the 4-profile model shows the highest classification accuracy, but the 4-profile model contained very small, unstable classes with proportions below 5%, threatening statistical robustness. The 2-profile model demonstrated balanced class proportions of 47.73 and 52.27%, both well above the recommended 5% threshold. In contrast, the 6-profile model contained very small unstable classes with proportions below 5%, seriously threatening statistical robustness and generalizability. Third, the two profiles identified in the 2-profile model—healthy adaptive eating and emotion-inhibited eating—were highly consistent with our theoretical framework based on the CEBQ, with clear conceptual boundaries. Fourth, the two classes in the 2-profile model demonstrated significant differences, exhibiting non-parallel qualitative differentiation patterns rather than mere differences in severity. The emotion-inhibited eating group scored significantly higher on “emotional undereating” and “food fussiness,” but significantly lower on “enjoyment of food” (see Figure 1). This multi-dimensional non-parallel pattern, rather than uniform high-low differences across all dimensions, supports the existence of qualitative differences (i.e., distinct types of eating behavior patterns) between the two profiles, rather than mere differences in severity. Based on the interpretability, theoretical significance, and parsimony criteria, the two-profile solution was retained.

**Figure 1:**
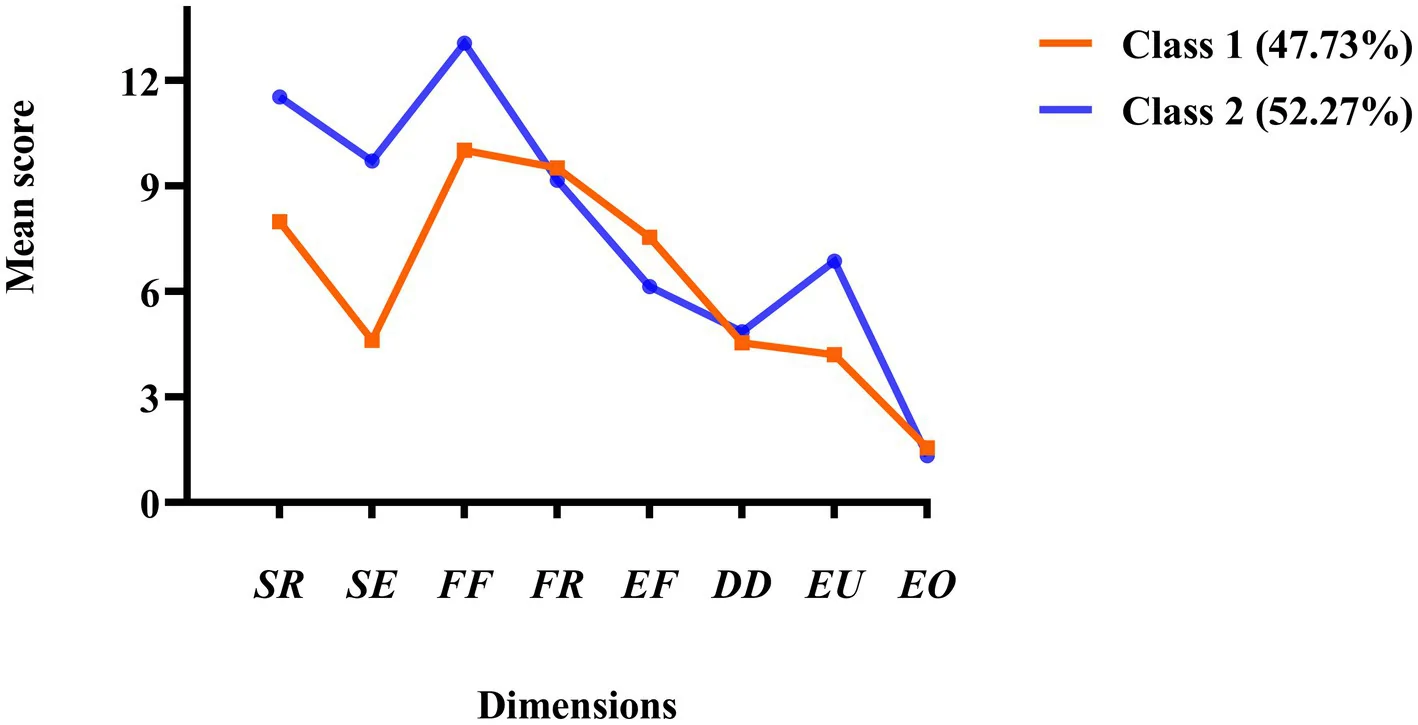
The characteristic distribution of 2 potential profiles of dietary behavior in pediatric patients with short stature. SR, Satiety responsiveness; SE, Slowness in eating; FF, Food fussiness; FR, Food responsiveness; EF, Enjoyment of food; DD, Desire to drink; EU, Emotional undereating; EO, Emotional overeating.

The CEBQ domain scores of the 2-profile model are presented in Figure 1 and Table 3. As evidenced in Figure 1, class C1 exhibited low scores for emotional undereating and emotional overeating, indicating that children’s eating is dominated by physiological signals, such as responses to satiety and proactive eating. Those in the class C1 were part of the “Healthy-adaptive eating group,” with 147 participants (47.73%). Those in class C2 exhibited low scores for emotional overeating but high scores for emotional undereating, revealing that emotions strongly influence children’s dietary behaviors. This group was named the “Emotion-inhibited eating group,” with 161 participants, and accounted for 52.27% of the 308 participants.

### 3.3 Comparison of differences in dietary behavior scores among pediatric patients with short stature

The Mann–Whitney U test was used to compare differences in dietary behaviors between the two groups. Notably, compared with the healthy adaptive eating group, the emotion-inhibited eating group received significantly higher scores on the dimensions of satiety responsiveness, slowness in eating, food fussiness, desire to drink, and emotional undereating, and, moreover, the differences were statistically significant (*p* < 0.05). However, no statistically significant difference was observed in the emotional overeating dimension scores between the two groups, as shown in Table 4.

**Table 4:** Differences in CEBQ scores among the latent profiles.

Domains	Healthy-adaptive eating group (*n* = 147)	Emotion-inhibited eating group (*n* = 161)	*Z*	*p*
Satiety responsiveness	8 (6, 10)	12 (9.5, 13)	−9.906	<0.001
Slowness in eating	4 (3, 6)	10 (8, 11)	−14.310	<0.001
Food fussiness	10 (8,12)	13 (11, 15)	−9.402	<0.001
Food responsiveness	10 (8, 11)	9 (8, 10)	−2.306	0.021
Enjoyment of food	7 (6, 9)	6 (5, 7)	−5.716	<0.001
Desire to drink	4 (4, 5)	5 (4, 5)	−2.035	0.042
Emotional undereating	4 (1, 6)	8 (4, 9)	−6.891	<0.001
Emotional overeating	1 (0, 3)	1 (0, 2)	−1.554	0.120

### 3.4 Univariate analysis of dietary behavior subtypes in pediatric patients with short stature

Univariate analysis revealed statistically significant differences between the two categories of dietary behavior in pediatric patients with short stature with respect to age, height SDS, BMI Z-score, and lack of pleasure (*p* < 0.05), as presented in Table 5.

**Table 5:** Influencing factors for the two latent dietary behavior profiles in pediatric patients with short stature.

Variables	Healthy-adaptive eating group (*n* = 147)	Emotion-inhibited eating group (*n* = 161)	χ^2^/*Z*	*p*
Gender			−0.753	0.452
Male	83 (56.46)	84 (52.17)		
Female	64 (43.54)	77 (47.83)		
Age (year)	9 (7, 11)	7 (6, 9)	−4.587	<0.001
Height SDS	−0.57 (−1.02, −0.05)	−1.19 (−1.55, −0.76)	−2.381	0.017
BMI Z-score	−0.94 (−1.49, −0.39)	−0.76 (−1.26, −0.25)	−2.502	0.012
School grade			6.304	0.074
Kindergarten	13 (8.85)	24 (14.91)		
Primary school	123 (83.67)	132 (81.99)		
Middle school	11 (7.48)	5 (3.10)		
Caregiver’s educational level			1.276	0.528
Primary school or below	16 (10.88)	23 (14.29)		
Junior high school	45 (30.61)	42 (26.09)		
Senior high school or above	86 (58.50)	96 (59.62)		
Family income			1.771	0.413
<3,000	14 (9.52)	9 (5.59)		
3,000 ~ 6,000	41 (27.89)	45 (27.95)		
>6,000	92 (62.59)	107 (66.46)		
Preterm birth			0.010	0.919
No	132 (89.80)	144 (89.44)		
Yes	15 (10.20)	17 (10.56)		
Children’s depression inventory				
Negative mood	2 (1, 3)	2 (1, 4)	−1.643	0.100
Low self-esteem	3 (2, 4)	3 (2, 4)	−0.435	0.663
Inefficacy	2 (1, 4)	3 (1, 4)	−1.855	0.064
Interpersonal difficulties	3 (1, 4)	3 (2, 4)	−1.367	0.172
Lack of pleasure	3 (2, 4)	4 (3, 6)	−3.188	0.001

### 3.5 Binary logistic regression analysis of dietary behavior subtypes in pediatric patients with short stature

The multicollinearity test revealed that the VIF values of all independent variables were < 5, indicating that there were no multicollinearity issues. Using the two latent dietary behavior profiles in pediatric patients with short stature as the dependent variable (with the healthy adaptive eating group as the reference), a binary logistic regression analysis was performed. Variables that indicated statistically significant differences in the univariate analysis, i.e., scores for age, height SDS, BMI Z-score, and lack of pleasure, were included as independent variables in the multivariate logistic regression model, and accordingly, these scores for age, height SDS, BMI Z-score, and lack of pleasure were entered as continuous observed values. The results showed that age (OR = 0.728, *p* < 0.001), Height SDS (OR = 0.738, *p* = 0.030), and lack of pleasure (OR = 1.239, *p* < 0.001) were independent influencing factors of emotion-inhibited eating. The results of the regression analysis are presented in Table 6.

**Table 6:** Binary logistic regression analysis of dietary behavior profiles in pediatric patients with short stature.

Project	*β*	SE	Wald χ^2^	OR (95%CI)	*p*
Content	1.588	0.562	7.973		0.005
Age (years)	−0.317	0.065	24.020	0.728 (0.641, 0.827)	<0.001
Height SDS	−0.304	0.140	4.738	0.738 (0.561, 0.970)	0.030
BMI Z-score	−0.157	0.083	3.627	0.854 (0.727, 1.005)	0.057
Lack of pleasure (score)	0.215	0.058	13.875	1.239 (1.107, 1.387)	<0.001

## 4 Discussion

The results of this study reveal that the dietary behavior score of pediatric patients with short stature (56.61 ± 9.79) was relatively high, indicating that dietary behavior problems are particularly prominent among this population, which is consistent with the findings of Xu L et al. (5). Additionally, the results of the LPA indicate significant heterogeneity in the dietary behaviors of pediatric patients with short stature, which can be classified into two categories: 47.73% of the pediatric patients with short stature were in the healthy-adaptive eating group, and 52.27% were in the emotion-inhibited eating group. Compared with the healthy-adaptive eating group, the emotion-inhibited eating group exhibited statistically significant differences (*p* < 0.05) in satiety responsiveness, slowness in eating, food fussiness, food responsiveness, enjoyment of food, desire to drink, and emotional undereating. These findings suggest that the dietary behaviors of pediatric patients with short stature are influenced by emotions.

The reasons for emotional eating among pediatric patients with short stature may involve complex interactions among physiological, psychological, and social factors. One study revealed that the rate of abnormal emotional behavior among children with short stature was 63.21%, which was significantly greater than that of children of normal height (27). Furthermore, school-age children with short stature exhibited significant negative psychological and emotional states, as well as low levels of psychological resilience (28). Owing to their short stature, children in school-age and adolescent transition periods are prone to feelings of inferiority and also tend to exhibit introverted personalities and significant emotional fluctuations. While emotional eating is one of the most common maladaptive coping strategies among adolescents (29), some cases of short stature are associated with insufficient growth hormone secretion or metabolic problems, which may indirectly affect appetite regulation and lead to abnormal appetites. Similarly, studies have concluded (30) that dopamine levels, leptin resistance, and serum cortisol levels are closely associated with emotional and restrictive eating. However, excessive concern about height among family members may also lead to control over the child’s diet or excessive compensation, thereby triggering an anxious relationship with food and rebellious eating behaviors. Given that children with short stature tend to eat slowly and exhibit picky eating habits, when parents frequently scold or intimidate children with short stature during meals, it creates a tense dining atmosphere (31) that often results in negative emotions in the child, a further reduction in food intake, and, ultimately, the exacerbation of dietary issues.

The results of this study indicate that 27.60% of the pediatric patients with short stature in the study population exhibited depressive symptoms, which is similar to the findings of Yan PP (32), who reported a prevalence of anxiety and depression of 26.01% among pediatric patients with short stature. There were no statistically significant differences among pediatric patients with short stature across different dietary behavior subtypes with respect to gender, school age, caregiver education level, household income, or preterm birth status. However, lack of pleasure was a significant predictor, distinguishing the emotional-inhibitory eating group from the healthy adaptive eating group (OR = 1.239, *p* < 0.001). These findings further suggest that anhedonia reduces the sensitivity of pediatric patients with short stature to the pleasurable aspects of eating, causing their eating motivation to shift toward alleviating negative emotions. As a result, pediatric patients with short stature are more likely to develop an inhibitory eating pattern oriented toward emotional regulation. Anhedonia is one of the core symptoms of adolescent depression; the inability to experience pleasure is an additional hedonic feature generated by the hedonic brain circuitry, primarily manifested as a loss of interest in or inability to derive enjoyment from daily activities (33). When children have poor eating habits or refuse to eat, some parents resort to scolding, punishment, or intimidation, thereby creating a tense mealtime atmosphere that reduces the child’s enjoyment of eating and leads to poor eating habits (31). This is attributed to the complex bidirectional interaction between children’s eating behaviors and negative emotions such as anxiety, depression, and perceived stress (34). On the one hand, negative emotions can exacerbate unhealthy eating behaviors; on the other hand, unhealthy eating behaviors can also affect emotional states, increasing the risk of depression and increasing depression symptom scores (8, 35), thereby creating a vicious cycle of negative emotions and unhealthy eating behaviors. Furthermore, this study revealed that height SDS and age were significantly negatively associated with emotion-inhibited eating among children. This finding is related to the fact that healthy eating behaviors can improve nutritional status and promote linear growth (12). In conclusion, clinical medical staff should attach great importance to the significant role of mental health in the management of the dietary behaviors of pediatric patients with short stature.

Researchers have reported that, compared with children of average height, children with short stature commonly experience significant negative emotions such as depression, low self-esteem, loneliness, and anxiety. Healthcare professionals should recognize the impact of emotional well-being on the eating behaviors of children with short stature (28, 36). Negative emotions may lead to reduced food intake and a lack of initiative to eat among these children. Parents should be advised to avoid coercion, criticism, scolding, and intimidation during meals. The use of positive reinforcement and rational emotive therapy helps enhance self-identity and alleviate negative emotions in children with short stature (37). Second, the family environment is crucial for the psychological health development of children. Inharmonious parent relationships may lead to tense family atmospheres, resulting in children who lack a sense of security and stable emotional support (38). A stable family environment and harmonious parent–child relationships can help reduce the psychological pressure associated with height issues for these children.

Therefore, when providing guidance on dietary behavior management for pediatric patients with short stature, clinical healthcare professionals should recognize the significant impact of emotional management on the development of healthy eating habits in this population. In addition to formulating balanced diet plans, it is essential that healthcare providers foster healthy attitudes toward food, avoid the use of food as a tool for emotional regulation, and guide families in maintaining harmonious relationships. Additionally, healthcare professionals should emphasize positive reinforcement through encouragement and praise, avoid placing excessive emphasis on height, and create an accepting environment to help pediatric patients with short stature develop healthy eating behaviors.

The limitations of this study are as follows: (1) The sample source was regionally restricted. This study investigated a tertiary-grade A hospital in Sichuan Province. Owing to geographical limitations and differences in dietary culture, it is necessary to recruit participants from different regions and ethnic groups in future studies to ensure the accuracy of the findings. (2) The prevalence estimate used for sample size calculation was derived from general child populations rather than specifically from pediatric patients with short stature. Currently, epidemiological studies specifically addressing eating behavior problems in pediatric patients with short stature remain scarce. Although we used 60% as the basis for estimation and our actual sample size exceeded the minimum required, the lack of precise prevalence data specific to this population may still affect the optimality of the sample size calculation. Future studies with more targeted epidemiological data are needed to further validate our findings. (3) The age distribution of our sample was markedly imbalanced, with children aged 6–11 years accounting for 90.3% of the participants and early adolescents aged 12–14 years comprising only 9.7%. This imbalance precluded robust age-stratified analyses, and thus the generalizability of our findings across developmental stages remains to be established in future studies with more balanced age distributions. (4) This study employed convenience sampling, which may introduce selection bias and affect the generalizability of the results. This study employed a cross-sectional design, which precluded the tracking of changes in the trajectories of emotion-inhibited eating among pediatric patients with short stature. Future research could adopt a longitudinal approach to explore the developmental patterns of dietary behaviors in this population, thereby further clarifying the causes of emotion-inhibited eating behaviors and identifying effective intervention strategies.

## 5 Conclusion

In conclusion, this study employed LPA and identified two distinct eating patterns among pediatric patients with short stature: healthy adaptive eating and emotion-inhibited eating. There are significant differences in the dietary behaviors of these two patterns, particularly with respect to emotional undereating and lack of pleasure. These findings suggest that healthcare professionals and caregivers should pay greater attention to the impact of emotions on the dietary behaviors of pediatric patients with short stature. Given the various characteristics of these children, precise dietary management strategies should be implemented to improve their poor dietary behaviors.

## Data Availability

The original contributions presented in the study are included in the article/supplementary material, further inquiries can be directed to the corresponding author.
